# Analysis of Cellular DNA Content in Pleural Effusion by Flow Cytometry During Lung Cancer Progression: A Case Report

**DOI:** 10.7759/cureus.76208

**Published:** 2024-12-22

**Authors:** Maria M Vasilescu, Marieta E Panait, Mirela Dumitru

**Affiliations:** 1 Department of Cancer Biochemistry and Radiobiology, Institutul Oncologic Prof. Dr. Alexandru Trestioreanu, Bucharest, ROU; 2 Department of Cancer Biology, Institutul Oncologic Prof. Dr. Alexandru Trestioreanu, Bucharest, ROU

**Keywords:** aneuploidy, cellular dna content, flow cytometry, lung cancer, pleural effusion

## Abstract

Malignant pleural effusion (MPE) is a common feature in patients with advanced or metastatic malignancies. While significant progress has been made in understanding the biology of pleural effusions, further research is needed to uncover the subsequent behavior of tumor cells following their invasion into the pleural space. This report utilizes flow cytometry to analyze DNA content abnormalities (aneuploidy) and cell cycle status, shedding light on the tumor cell populations present in MPE samples from a patient with lung adenocarcinoma during treatment. The findings suggest that under selective pressure, certain tumor cell subpopulations within the pleural effusion were suppressed, while therapy-resistant subpopulations emerged, driving disease progression. MPE serves as a valuable model for studying tumor heterogeneity and clonal dynamics in real time, offering insights that may inform diagnosis, prognosis, and therapeutic strategies.

## Introduction

Malignant pleural effusion (MPE), defined as the presence of malignant cells in pleural fluid, arises due to direct or indirect involvement of the pleura [1]. MPE is observed in 8-15% of patients at initial evaluation and in 40-50% during disease progression. The most frequent cause of MPE is lung cancer, followed by breast cancer, lymphoma, gynecological malignancies, mesothelioma, and cases with unknown primary sites. Interestingly, only 55-60% of patients with pleural metastasis develop MPE [2]. Adenocarcinoma, the most common subtype of non-small cell lung cancer (NSCLC), is frequently associated with MPE, and its presence often indicates an advanced disease stage or progression [3].

For decades, cytological examination of pleural fluid has been the first-line diagnostic tool for pleural effusion and suspected malignancy. It remains widely used, with significant implications for determining subsequent investigations. However, the sensitivity of cytological examination for detecting malignancy varies widely, ranging from 40% to 87% [4]. To address these limitations, new methods have been introduced to enhance the identification of tumor cells and improve the accuracy of conventional cytological diagnosis. Among these, flow cytometry has demonstrated utility in analyzing body fluids such as pleural effusion, effectively distinguishing between normal and malignant cell populations [5,6].

In this study, we explored the cellular heterogeneity within MPE samples from a patient with lung adenocarcinoma. Using flow cytometry analysis of DNA content abnormalities (aneuploidy) and cell cycle status, we assessed the tumor cell populations in pleural fluid over the course of treatment. This approach enabled us to monitor the dynamics of tumor subpopulations under selective pressure, providing valuable insights into disease progression.

## Case presentation

A 38-year-old female non-smoker presented to our hospital with right-sided chest pain, loss of appetite, severe dyspnea, and weight loss. The patient had no history of significant comorbidities. Clinical examination revealed right-sided pleural effusion syndrome, and blood tests showed anemia and an increased erythrocyte sedimentation rate (Table 1). The diagnosis was a right hilar lung tumor (adenocarcinoma) with right pleurisy due to adenocarcinoma metastasis.

**Table 1:** Patient’s blood tests at presentation

Parameters (units)	Values	Reference range
Hemoglobin (g/dL)	10.8	12-14
Hematocrit (%)	36	36-48
Erythrocyte sedimentation rate (mm/h)	44	<22
Glucose (mg/dL)	83	70-115
Glutamic oxaloacetic transaminase (U/L)	4	<34
Glutamic pyruvic transaminase (U/L)	4	<55
Urea (mg/dL)	20	15-40

The patient received five consecutive cycles of polychemotherapy, including platinum compounds (Paraplatin), gemcitabine (Gemzar), taxanes (Taxotere), vinca alkaloid (Eldesine), alpha interferons (INTRON A), and antiestrogens (Tamoxifen). Iscador, an extract of *Viscum album*, was used as an adjuvant to chemotherapy. Following the initiation of chemotherapy, therapeutic thoracenteses and intracavitary instillations of thiotepa, an alkylating agent, were performed during each round of treatment to prevent the recurrence of pleural effusion. The patient’s medical condition and blood count parameters at the time of thoracentesis are shown in Table 2 and Table 3, respectively.

**Table 2:** Medical condition at the time of thoracentesis

Thoracentesis (number)	Medical condition at the time of thoracentesis
I	Hilar lung tumor with right metastatic adenocarcinoma pleurisy; profound dyspnea
II	Condition slightly improved
III	Alteration of general condition; dyspnea with orthopnea; pulmonary metastases
IV	Moderate condition; pain at the right hemithorax
V	Stationary condition; bone (costal) metastases
VI	Alteration of the general condition

**Table 3:** Blood count parameters at the time of thoracentesis

Parameters (units)	Values						Reference range
	Thoracentesis (number)						
	I	II	III	IV	V	VI	
Hemoglobin (g/dL)	11	12.2	12.4	11.2	10.8	8.6	12-14
White blood cell (× 10^3^/µL)	6	7.8	4.2	5	7.4	4	5-10
Platelet count (× 10^3^/µL)	150	150	160	190	210	170	150-450
Neutrophils, absolute (× 10^3^/µL)	3	5.4	0.8	1.5	4.6	1.2	2.5-7.5
Eosinophils, absolute (× 10^3^/µL)	0.3	0.2	0.2	0.3	0.2	0.3	0.02-0.5
Basophils, absolute (× 10^3^/µL)	0	0	0.1	0	0	0	0.0-0.2
Lymphocytes, absolute (× 10^3^/µL)	2	2	2.5	2.7	2	1.8	1-4
Monocytes, absolute (× 10^3^/µL)	0.7	0.4	0.6	0.5	0.6	0.7	0.1-1.3

**Figure 1:**
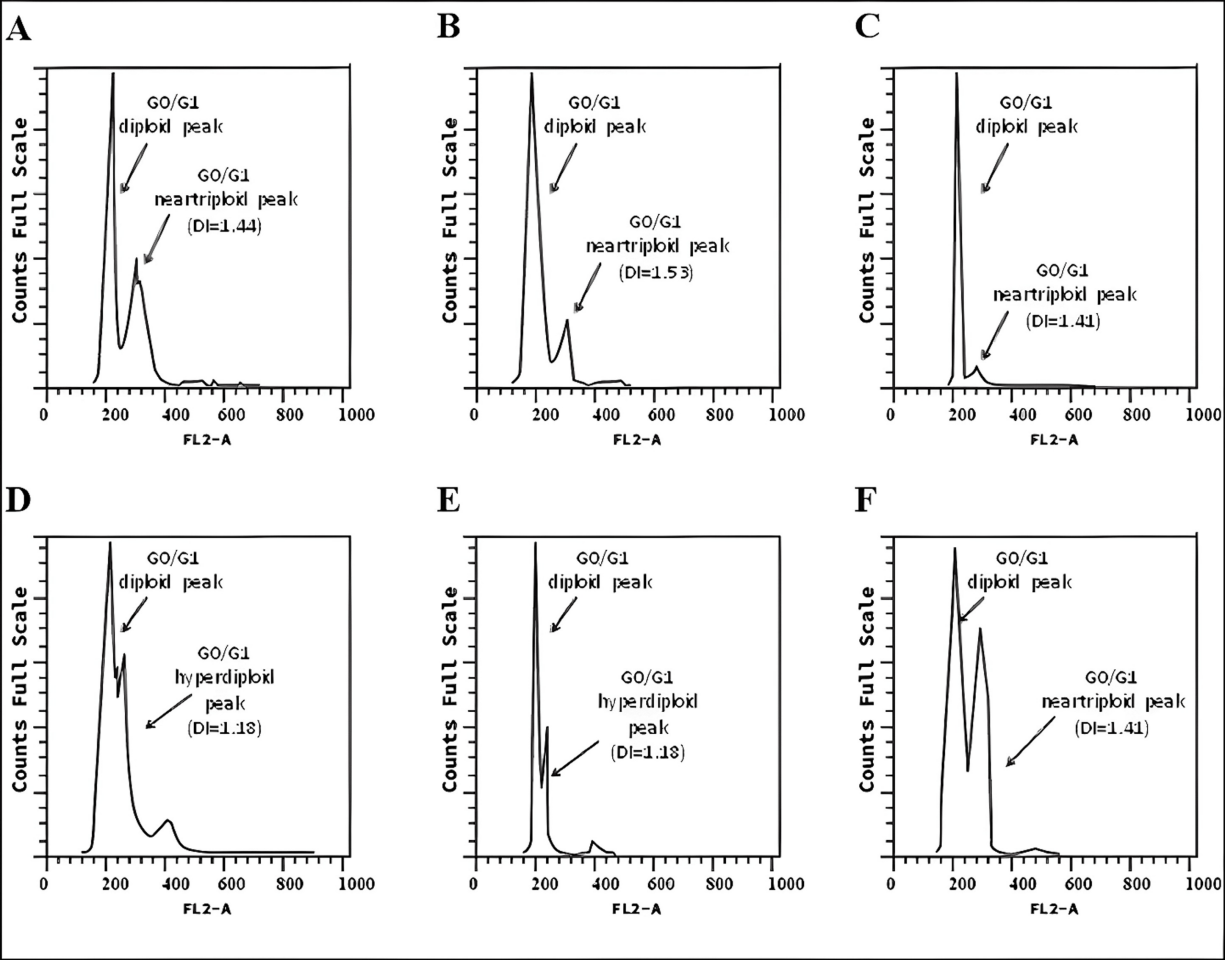
Cell cycle distribution in MPE analyzed by flow cytometry (A) Cell cycle histogram profile of pleural effusion before therapy initiation. (B) Cell cycle histogram profile of pleural effusion one month after the first therapy administration. (C) Cell cycle histogram profile of pleural effusion one month after the second round of treatment. (D) Cell cycle histogram profile of pleural effusion one month after the third round of treatment. (E) Cell cycle histogram profile of pleural effusion one month after the fourth round of treatment. (F) Cell cycle histogram profile of pleural effusion one month after the fifth round of treatment. MPE, malignant pleural effusion

Six serial samples of recurrent pleural effusion, collected throughout the course of treatment, were analyzed for cellular DNA content and cell cycle phases by flow cytometry. The pleural effusion samples were obtained via thoracentesis before the start of each round of treatment. The percentage of cell populations, DNA ploidy (expressed as DNA index, DI), and cell cycle distribution of pleural fluid cells were determined from the single-parameter histograms (Figure 1).

**Figure 2:**
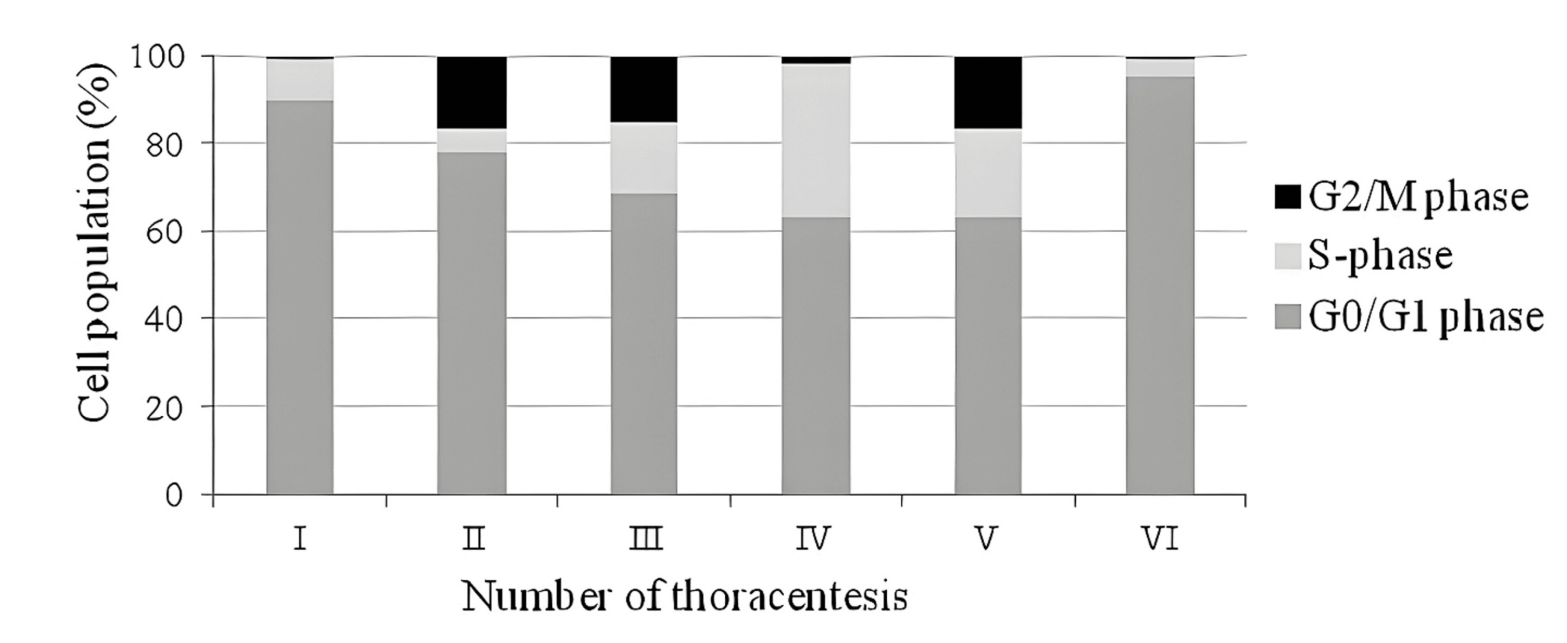
Quantitative cell cycle analysis of aneuploid populations

DNA ploidy analysis of the specimen collected before the initiation of therapy (Figure 1A) revealed a tumor cell population with abnormal DNA content, identified by a near-triploid peak (DI = 1.44) in the DNA histogram. This population comprised 42.9% of cells, with 89.7% in the G0/G1 phase and 9.6% in the S-phase. One month after the first combination therapy (Figure 1B), the near-triploid tumor population (DI = 1.53), representing 20.4% of cells, showed a decrease in the percentage of cells in G0/G1 and S-phases, accompanied by a simultaneous G2/M phase arrest. After another month of treatment (Figure 1C), a much smaller near-triploid population (DI = 1.41), representing 14% of cells, exhibited increased S-phase and G2/M phase proportions compared to previous analyses. The following month (Figure 1D), an aneuploid population with slightly hyperdiploid DNA content (DI = 1.18) became dominant, comprising 51.7% of the cells. This population showed 34.8% in the S-phase and 2.1% in the G2/M phase. The analysis conducted after four months of treatment (Figure 1E) revealed a decrease in the hyperdiploid population to 41%, with 20% in the S-phase and 16% in the G2/M phase. One month later (Figure 1F), tumor populations shifted, with a near-triploid population (DI = 1.44) characterized by lower S-phase and higher G0/G1 phase proportions. The final analysis of pleural fluid showed a cell cycle profile similar to that observed before treatment. The percentage distribution of cell cycle phases within aneuploid populations for each of the six serial pleural effusion samples is summarized in Figure 2.

At the time of the sixth thoracentesis, the patient presented with anemia, neutropenia, and leukopenia (Table 3). Unfortunately, after five rounds of treatment, the patient showed no favorable clinical outcome and did not return for further treatment.

## Discussion

The management of MPE remains a topic of debate, and the lack of effective treatment options underscores the need for a deeper understanding of the basic biological aspects of pleural metastasis and malignant effusion accumulation. The coexistence of diverse clonal populations of tumor cells is characteristic of both primary tumors and metastases [7]. Tumors are dynamic, evolving entities, and clonal heterogeneity can vary spatially and temporally. As the disease progresses, tumors may exhibit differences in their clonal composition. Various factors, including the acquisition of driver mutations and selective pressures from the microenvironment or therapeutic interventions, can influence tumor heterogeneity [8]. Several studies have shown that drug-resistant subpopulations can exist prior to therapy and may expand during treatment. For example, during sequential monotherapy with epirubicin and docetaxel in primary breast cancer, smaller subclones redistributed, while major subclones persisted throughout treatment [9]. Schwarz et al. found that subclonal tumor populations in high-grade serous ovarian cancer pre-treatment biopsies continued to evolve during chemotherapy, ultimately leading to relapse [10].

In line with these findings, our case study revealed that under chemotherapy, certain subpopulations of tumor cells from pleural effusion were suppressed, while therapy-resistant subpopulations dominated, leading to disease progression. Previous studies on DNA ploidy in lung tumors and pleural effusions have shown a high level of aneuploidy in lung cancer [11-14]. Aneuploidy occurs in approximately 60% of NSCLCs, and in small-cell lung cancer, the incidence is 77.8% [15]. Overall, evidence suggests that MPEs predominantly exhibit aneuploid DNA content, while benign effusions typically present with euploid DNA [16].

Aneuploidy can contribute to the development and selection of malignant clones, driving phenotypic diversification during tumor progression [15]. In their review of clinical applications for aneuploidy in NSCLC, Yan et al. proposed aneuploidy as a new biomarker, with multiple studies linking high levels of aneuploidy to increased invasiveness, poor prognosis, and metastasis in NSCLC [15]. A strong association has also been observed between the degree of aneuploidy and resistance to chemotherapy, particularly with drugs that inhibit cell division. This association is also present with non-cell cycle-specific chemotherapies [17]. For patients with malignant involvement of the serous cavities, combined systemic chemotherapy with drugs acting through different mechanisms may improve survival outcomes. The patient in this study received such treatment but still experienced unfavorable progression. Analysis of cellular DNA content in MPE samples revealed the maintenance of aneuploid status throughout treatment, which was associated with metastases at different sites and resistance to treatment. Chromosomal instability due to aneuploidy influences therapeutic outcomes under selective pressure, such as chemotherapy regimens, promoting the survival of subclones that acquire resistance to specific chemotherapeutics [18,19].

A key aspect of this study is that DNA content analysis was conducted using only flow cytometry, which considered only the DNA content of the dominant cell population and not the morphology of the cells. Additionally, the small size of the available tissue biopsy limited the ability to perform further tests, such as immunohistochemistry.

## Conclusions

The presence of tumor populations with aneuploid DNA is a known indicator of aggressiveness in tumor lesions and is associated with a poorer prognosis in most cases. While our case report is not the first to utilize pleural effusion to detect aneuploidy in patients with NSCLC, it provides unique insight into the variation of dominant tumor subpopulations during chemotherapeutic intervention in MPE samples from a lung adenocarcinoma patient. This was achieved through flow cytometry analysis of DNA content abnormalities (aneuploidy) and cell cycle status. Our findings support the use of ploidy analysis as a valuable tool for monitoring tumor progression. Future studies should confirm aneuploidy through methods such as FISH, PCR, or image cytometry. MPE may serve as a valuable model for studying tumor heterogeneity and clonal dynamics in real time. The presence of cellular heterogeneity poses a significant challenge in cancer therapy, as various subpopulations can evolve in response to treatment, leading to dynamic heterogeneity and, ultimately, resistance to therapy.
